# Long noncoding RNA PSMA3-AS1 functions as a competing endogenous RNA to promote gastric cancer progression by regulating the miR-329-3p/ALDOA axis

**DOI:** 10.1186/s13062-023-00392-8

**Published:** 2023-07-04

**Authors:** Liang Kan, Meiqi Yang, Huijing Zhang

**Affiliations:** 1 Department of Geriatrics, Shengjing Hospital of China Medical University, Shenyang, 110004 China; 2 Department of Endoscopy, The First Affiliated Hospital of China Medical University, 155 North Nanjing Street, Shenyang, 110001 China

**Keywords:** lncRNA PSMA3-AS1, Gastric cancer, miR-329-3p, ALDOA, cancer progression

## Abstract

LncRNA PSMA3-AS1 functions as an oncogene in several cancers, including ovarian cancer, lung cancer, and colorectal cancer. However, its role in gastric cancer (GC) progression remains unclear. In this study, the levels of PSMA3-AS1, miR-329-3p, and aldolase A (ALDOA) in 20 paired human GC tissues and adjacent nontumorous tissues were measured by real-time PCR. GC cells were transfected with recombinant plasmid carrying full-length PSMA3-AS1 or shRNA targeting PSMA3-AS1. The stable transfectants were selected by G418. Then, the effects of PSMA3-AS1 knockdown or overexpression on GC progression in vitro and in vivo were evaluated. The results showed that PSMA3-AS1 was highly expressed in human GC tissues. Stable knockdown of PSMA3-AS1 significantly restrained proliferation/migration/invasion, enhanced cell apoptosis, and induced oxidative stress in vitro. Tumor growth and matrix metalloproteinase expression in tumor tissues were markedly inhibited, while oxidative stress was enhanced in nude mice after stable PSMA3-AS1 knockdown. Additionally, PSMA3-AS1 negatively regulated miR-329-3p while positively regulated ALDOA expression. MiR-329-3p directly targeted ALDOA-3′UTR. Interestingly, miR-329-3p knockdown or ALDOA overexpression partially attenuated the tumor-suppressive effects of PSMA3-AS1 knockdown. Conversely, PSMA3-AS1 overexpression exhibited the opposite effects. PSMA3-AS1 promoted GC progression by regulating the miR-329-3p/ALDOA axis. PSMA3-AS1 might serve as a promising and effective target for GC treatment.

**Supplementary Information:**

The online version contains supplementary material available at 10.1186/s13062-023-00392-8.

## Introduction

Gastric cancer (GC) is the fourth most common cancer among men and the seventh among women [1, 2]. In 2020, it contributes to 1.09 million new cases and 0.77 million deaths [1]. GC incidence is two-fold higher in men [3]. Several risk factors are identified, including *Helicobacter pylori* infection, family history, low socioeconomic status, physical inactivity, Epstein-Barr virus infection, high-salt diet, and smoking [4, 5]. To date, surgical resection combined with perioperative chemotherapy, radiation therapy, immunotherapy, or targeted therapy is still the standard treatment regimen for GC [6]. Despite the improvements in GC treatment, patients′ prognosis remains poor [7]. Therefore, elucidating the pathogenesis of GC will help us to explore more effective therapeutic strategies.

Long noncoding RNAs (lncRNAs) play essential roles in numerous biological processes, including tumorigenesis, autophagy, apoptosis, cell differentiation, epigenetic control, and embryonic development [8]. LncRNA PSMA3-AS1 is overexpressed in numerous cancers, e.g. oral squamous cell carcinoma, lung cancer, colorectal cancer, ovarian cancer, and esophageal squamous cell carcinoma [9–13]. A previous study has revealed that PSMA3-AS1 facilitates growth/metastasis of esophageal cancer [10]. Conversely, Huang et al. demonstrated that PSMA3-AS1 knockdown suppressed glioma cell proliferation and promoted apoptosis [14]. In addition, PSMA3-AS1 knockdown could also restrain cell migration and invasion in other human cancers [12, 13]. Nevertheless, the role of PSMA3-AS1 in GC is still unknown.

MicroRNAs (miRNAs) participate in the development of cancers, serving as oncogenes or tumor suppressors [15]. Mounting evidence has suggested that miR-329-3p is downregulated in many human cancers [16–19], including in GC [20]. Low miR-329-3p level in cervical cancer correlates with poor prognosis [16, 21]. Another research by Xin et al. reported that miR-329-3p restrained hepatocellular carcinoma cell proliferation and migration [22]. Nevertheless, the relationship between miR-329-3p and PSMA3-AS1 in GC remains unknown.

In our study, bioinformatics analysis predicated the relationship between miR-329-3p and PSMA3-AS1 or ALDOA. Our results demonstrated that PSMA3-AS1 facilitated GC progression via upregulation of ALDOA by targeting miR-329-3p.

## Materials and methods

### Patients and specimens

Human fresh GC tissues and adjacent nontumorous tissues were obtained from 20 patients from March 2019 to June 2021 in the First Affiliated Hospital of China Medical University. None of the patients underwent preoperative chemotherapy or radiotherapy. The studies involving human participants were reviewed and approved by the Ethics Committee of the First Affiliated Hospital of China Medical University. The patients/participants provided their written informed consent to participate in this study.

### Cell lines

Normal gastric epithelial GES-1 cells, GC cell lines (HGC-27, AGS, NCI-N87, and SNU-1), and HEK-293T cells were obtained from Procell (Wuhan, China). AGS and HEK-293T cells were maintained in Ham′s F-12 medium (Gibco, Shanghai, China) and DMEM, respectively, supplemented with 10% FBS (Gibco). Meanwhile, RPMI-1640 (Gibco) containing 20% FBS was used to culture HGC-27 cells. GES-1, NCI-N87, and SNU-1 cells were grown in RPMI-1640 containing 10% FBS at 37 °C with 5% CO_2_.

### Stable transfectants

The full-length PSMA3-AS1 and ALDOA were synthesized by GenScript (Nanjing, China) and inserted into pcDNA-3.1 vector (Invitrogen) between the *Hin*d III and *Bam*H I sites. pRNA-H1.1 vectors carrying shRNA against PSMA3-AS1 (sh-PSMA3-AS1) and control shRNA (sh-NC) between the *Hin*d III and *Bam*H I sites were constructed by GenePharma (China). The recombinant plasmids containing sh-PSMA3-AS1 and full-length PSMA3-AS1 were transfected into AGS and HGC-27 cells, respectively, using Lipofectamine 2000 (Invitrogen). The stable transfectants were selected by G418 (400 µg/ml) for 6 weeks.

### Cell transfection

To investigate the effect of miR-329-3p on ALDOA expression, AGS and HGC-27 cells were transfected with miR-329-3p mimic or inhibitor (50 nM; GenePharma) using Lipofectamine 2000 (Invitrogen). Forty-eight hours post-transfection, ALDOA expression was examined by real-time PCR. For rescue experiments, miR-329-3p inhibitor (50 nM) or ALDOA overexpression plasmid (500 ng) was transfected into AGS cells with stable PSMA3-AS1 knockdown using Lipofectamine 2000 (Invitrogen).

### Real-time PCR and droplet digital PCR (ddPCR)

The cells and tissues were homogenized in TRIzol reagent (Invitrogen) to isolate total RNA, and then the total RNA was quantified by an ultraviolet spectrophotometer (Biochrom, Cambridge, UK). After that, RNA was reverse-transcribed into cDNA using M-MLV reverse transcriptase (Beyotime, Haimen, China). For real-time PCR, amplification reaction was conducted on a Real-time PCR system (SLAN-96 S; Shanghai Hongshi Medical, China) using SYBR Green qPCR Mix (Beyotime). Thermal cycling conditions were as follows: 95 °C for 10 min, followed by 40 cycles of 95 °C for 10 s, 60 °C for 20 s, and 72 °C for 30 s. The relative levels were calculated using 2^−ΔΔCt^ method. GAPDH served as an internal control for mRNA and lncRNA, and U6 served as an internal control for miRNA. The primers used in this study are listed in Table 1. ddPCR was performed using the QX200 ddPCR system (Bio-Rad, USA). Droplets were generated for each PCR reaction mixture using droplet generation oil for probes, and then they were transferred to 96-well plates. Thermal cycling conditions were as follows: 95 °C for 10 min, 40 cycles at 94 °C for 30 s and 60 °C for 1 min, followed by 98 °C for 10 min. After that, the droplets were read using the QX200™ Droplet Reader, and the data were analyzed using Quantasoft™ version 1.7.4 software.

**Table 1:** Primer sequences for Real-time PCR

Gene	Forward (5′-3′)	Reverse (5′-3′)
PSMA3-AS1	AACAGACCATCAGAAGAGAACA	GAACAGAAACCAGAGCCATACA
miR-329-3p	GTGGAACAGACCTGGTAAAC	CAAGTGCGAGTCGTGCAGT
ALDOA	ATGCCCTACCAATATCCAGC	GACAGCCCATCCAACCCT
U6	GCTTCGGCAGCACATATACT	GTGCAGGGTCCGAGGTATTC
GAPDH	GACCTGACCTGCCGTCTAG	AGGAGTGGGTGTCGCTGT

### CCK-8 assay

The cells were plated onto 96-well plates at 3000 cells/well. Then, the plates were placed in a 5% CO_2_ incubator and cultured for 0 − 72 h. Afterwards, the supernatant was aspirated and culture medium (100 µl) was added into each well. CCK-8 reagent (10 µl) (Solarbio) was used to treat the cells for 1 h at 37 °C. The optical density at 450 nm was monitored.

### Colony formation assay

The cells were harvested by centrifugation after trypsin incubation and then seeded on 6-well plates (500 cells/well). The cells were allowed to grow for 2 weeks at 37 °C to form visible colonies. After three PBS washes, the fixed cells were incubated with crystal violet (ASPEN, Wuhan, China) for 30 min. Colony numbers were counted by microscopy (OLYMPUS). Colony formation rate was calculated using the following formula: Colony formation rate = (numbers of colonies / numbers of seeded cells) × 100%.

### Cell cycle analysis by flow cytometry

The GC cells were trypsinized to prepare a single-cell suspension. Then, the cells were washed once and resuspended in PBS (100 µl). Precooled ethanol (300 µl) was gently added to fix the cells for 24 h. Subsequently, they were washed once in PBS, and resuspended in a buffer containing propidium iodide and RNase A (10 µl of each) (YEASEN, Shanghai, China). After 30 min of incubation, a flow cytometer (CytoFLEX; Beckman, Brea, CA, USA) was used to assess cell cycle.

### Cell apoptosis by flow cytometry

The GC cells were trypsinized and centrifugated at 300×g for 5 min at 4 °C. After twice PBS washes in precooled PBS and centrifugation, the cells were resuspended in a binding buffer (300 µl) containing 5 µl of Annexin V-FITC (Sungene, Tianjin, China) for 10 min in the dark. The cells were further stained with 5 µl of propidium iodide (PI; Sungene) for 5 min, and then subjected to flow cytometry (CytoFLEX; Beckman). Annexin V^–^/PI^–^ cells represent viable cells. Annexin V^+^/PI^–^ cells represent early apoptotic cells. Annexin V^+^/PI^+^ cells represent late apoptotic cells. Annexin V^–^/PI^+^ cells represent necrotic cells. Early and late apoptotic rates were assessed using FlowJo software (Ashland, OR, USA). The apoptotic rate was determined by counting early and late apoptotic rates.

### Western blotting

The GC cells and tumor tissues were lysed, and total proteins were extracted. Protein extracts (20 µg) were separated by 12% SDS-PAGE (at 80 V for 40 min followed by at 120 V for 50 min). After electrophoresis, proteins were transferred to PVDF membranes at 90 V for 50 min. Then, PVDF membrane was exposed to 3% BSA for 30 min and then reacted with an antibody against ALDOA (1:2000 dilution, Abcam, Shanghai, China), cleaved caspase-3 (1:1000 dilution, Affinity Biosciences, Liyang, China), cleaved PARP (1:1000 dilution, CST, Shanghai, China), MMP-2 (1:500 dilution, Abcam), MMP-9 (1:1000 dilution, Abcam), Bax (1:1000 dilution, CST), Bcl-2 (1:2000 dilution, Abcam), CDK6 (1:2000 dilution, Proteintech, Wuhan, China), or cyclin D1 (1:1000 dilution, CST) at 4 °C overnight. Afterwards, the membrane was exposed to a secondary antibody (1:10000 dilution, ASPEN) for 30 min at 37 °C. The bands were visualized and analyzed by NIH ImageJ software.

### Wound healing assay

A total of 5 × 10^5^ cells in 2 ml culture medium were seeded on 6-well plates. After adhering to the plates, the cell monolayer was scratched and cell debris was removed by a wash with PBS. The cells were further cultured for 24 and photographed using an inverted microscope (IX51; OLYMPUS). Wound healing rate (%) was calculated using the following formula to assess cell migratory capability: Wound healing rate (%) = (initial wound width at 0 h – wound width at 24 h) / initial wound width at 0 h × 100%.

### Transwell assay

The lower and upper chambers of the Matrigel-coated insert were filled with 10% FBS-containing medium (500 µl) and cell suspension containing 2 × 10^4^ cells, respectively. After 48 h of culture, the cells were stained with crystal violet (ASPEN) for 10 min. The numbers of invaded cells were counted in five random fields using an inverted microscope (IX51; OLYMPUS), and the average numbers of invaded cells were calculated.

### Measurements of intracellular ROS, MDA content, GSH-Px activity, and SOD activity

Intracellular ROS level was monitored using a ROS assay kit (Beyotime). Briefly, the cells were harvested, and then they were exposed to DCFH-DA (10 µM) for 20 min. After three washes and resuspension in PBS, intracellular ROS was determined by using a fluorescence plate reader (M200Pro; Tecan, Switzerland). MDA content as well as activities of GSH-Px and SOD in both GC cells and tumor tissues were examined using commercial kits (Jiancheng, Nanjing, China) according to the manufacturer′s instructions.

### Immunofluorescence

The cells on coverslips were fixed in paraformaldehyde (4%) for 20 min and washed three times with PBS. The cells were reacted with anti-Nrf2 antibody (1:200 dilution, Proteintech) at 4 °C overnight, and then incubated with Cy3-conjugated anti-rabbit IgG (1:100 dilution, ASPEN) for 40 min at 37 °C. After 30 min of nuclei staining with DAPI, the coverslips were examined by fluorescence microscopy (OLYMPUS).

### Dual-luciferase reporter assay

HEK-293T cells were allowed to grow to approximately 70% confluence, and then they were starved for 1 h. Afterwards, the cells were co-transfected with miR-329-3p mimic/NC mimic (50 nM) and recombinant plasmid carrying wild-type/mutant PSMA3-AS1 (sites 1 and 2) or ALDOA-3′UTR (100 ng) using Lipofectamine 2000 (Invitrogen). Forty-eight hours post-transfection, the firefly luciferase activity was examined using Dual-Luciferase reporter assay kit (Promega, Beijing, China) with a M200Pro microplate reader (Tecan) and normalized to the luciferase activity of Renilla.

### Tumor xenografts in nude mice

Male nude mice (5- to 6-week-old) were allotted to 6 groups, and each group contained 6 mice. After 1 week of adaptive feeding, the nude mice were subcutaneously injected with 1 × 10^6^ stable transfectants. Long and short diameters of tumors were measured every 4 days to calculate tumor volumes (V = 1/2 × L × S^2^). The mice were euthanatized, and tumors were excised and weighed on day 32 after the first injection. The animal study was performed in accordance with Guide for the Care and Use of Laboratory Animals and was approved by the Institutional Animal Care and Use Committee of China Medical University.

### Statistical analysis

Data are expressed as mean ± SD. Student′s t-test was used to compare two groups, and one-way ANOVA with Tukey′s post-hoc test was used to compare multiple groups. *P* < 0.05 was considered statistically significant.

## Results

### PSMA3-AS1 is highly expressed in both GC tissues and cell lines

**Fig. 1:**
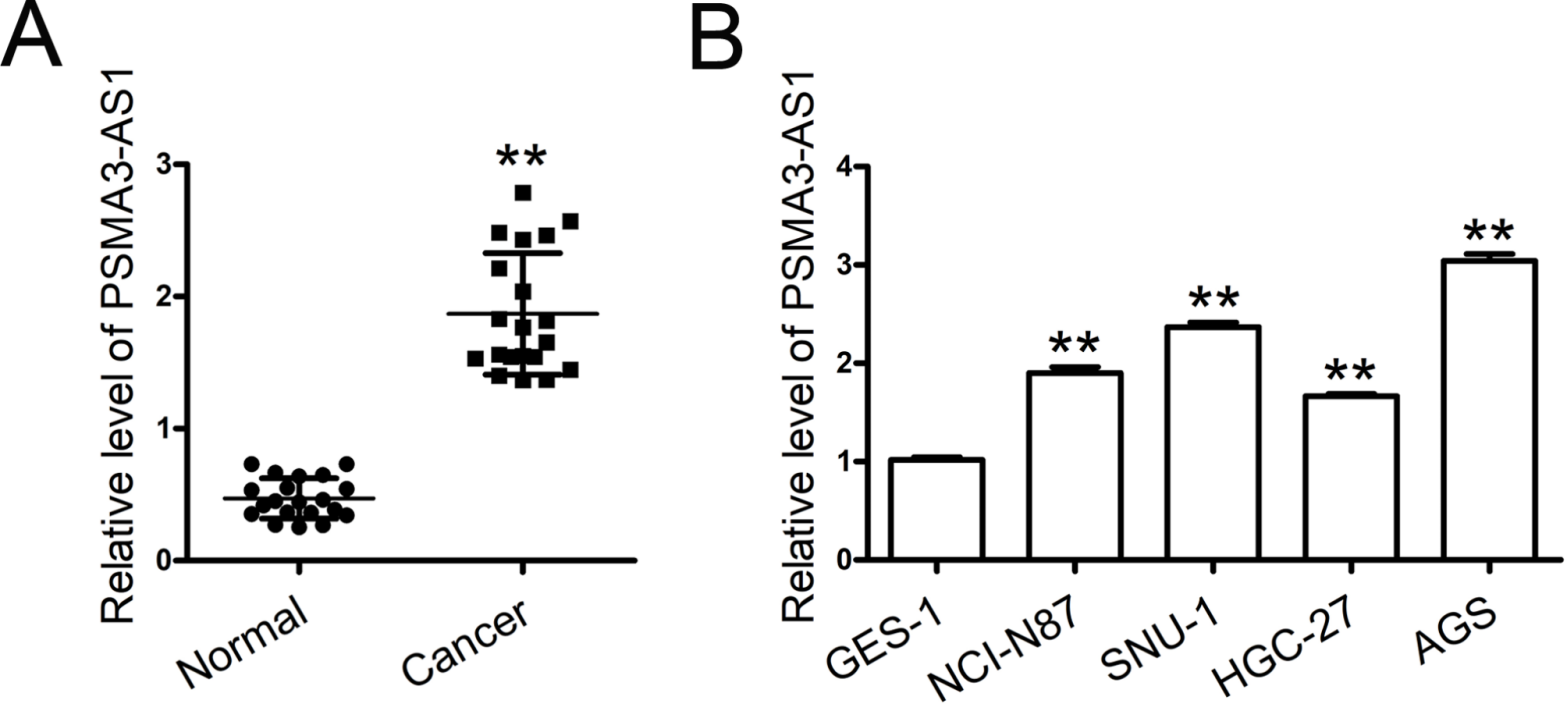
PSMA3-AS1 is upregulated in GC tissues and cell lines. **(A)** Human fresh tumor tissues and adjacent nontumorous tissues were obtained from patients with GC. PSMA3-AS1 levels in 20 paired clinical specimens were examined by real-time PCR (n = 20). GAPDH served as an internal control. **(B)** PSMA3-AS1 levels in four GC cell lines and GES-1 cells were determined by real-time PCR (n = 3). GAPDH served as an internal control. ^**^P < 0.01 compared with Normal or GES-1 cells. Student′s t-test was used to compare two groups, and one-way ANOVA with Tukey′s post-hoc test was used to compare multiple groups

We examined PSMA3-AS1 levels in 20 paired clinical specimens. PSMA3-AS1 was greatly elevated in GC tissues compared to that in adjacent normal tissues (Fig. 1A). Additionally, all four GC cell lines expressed higher PSMA3-AS1 than gastric epithelial cells (Fig. 1B). AGS cells had a higher level of PSMA3-AS1 and HGC-27 cells had a lower one than other GC cells. Thus, AGS and HGC-27 cells were selected to knock down and overexpress this lncRNA, respectively.

### PSMA3-AS1 knockdown restrains cell proliferation, migration, and invasion of GC cells

**Fig. 2:**
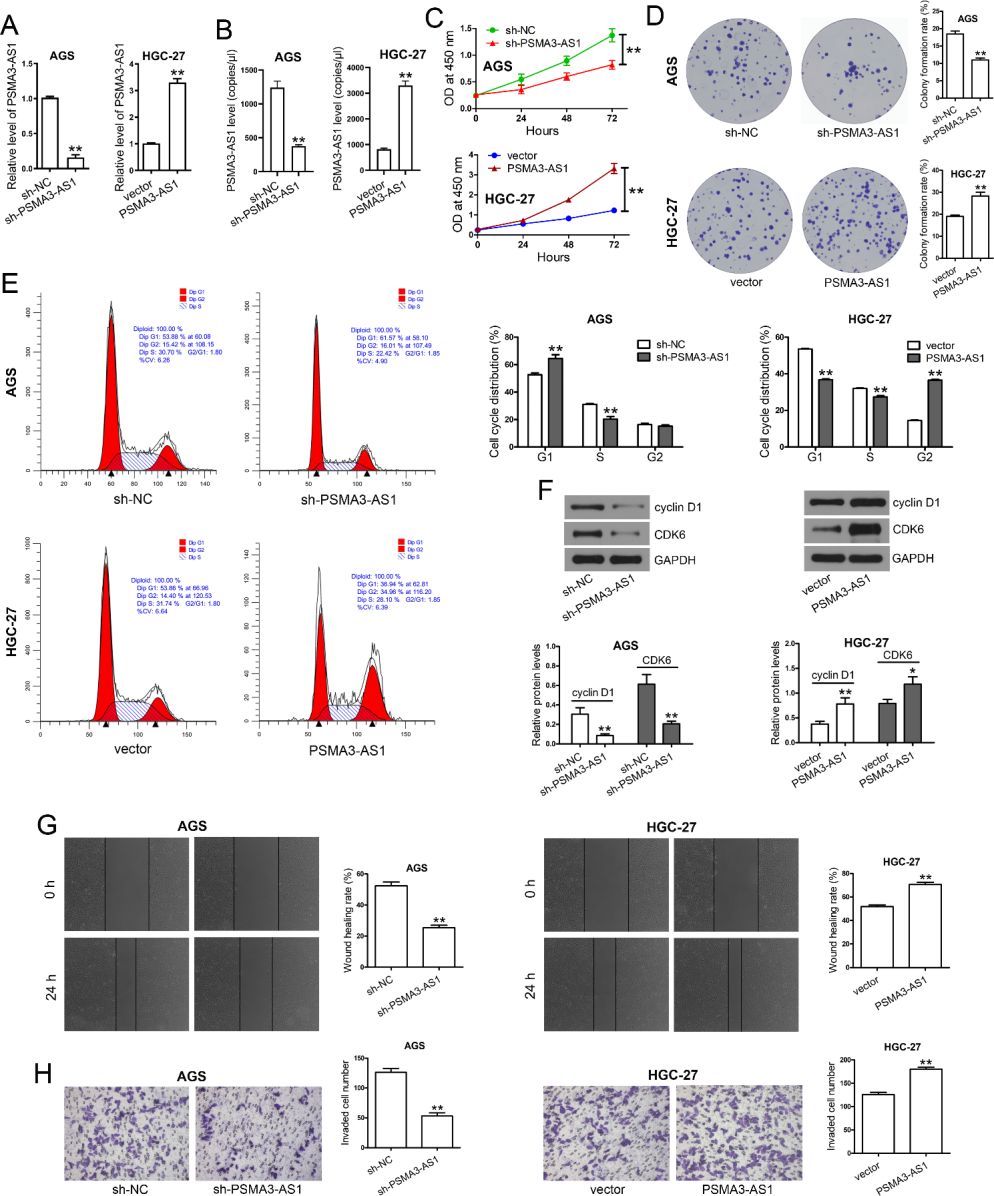
PSMA3-AS1 knockdown restrains cell proliferation, migration, and invasion of GC cells. **(A)** A pcDNA-3.1 vector containing full-length PSMA3-AS1 and a pRNA-H1.1 vectors carrying sh-PSMA3-AS1 were constructed via the *Hin*d III and *Bam*H I sites. PSMA3-AS1 overexpression plasmid was transfected into HGC-27 cells, while PSMA3-AS1 knockdown plasmid was transfected into AGS cells using Lipofectamine 2000. The stable transfectants were selected by G418 (400 µg/ml) for 6 weeks. PSMA3-AS1 levels in stable transfectants were measured by real-time PCR (n = 3). GAPDH served as an internal control. **(B)** The copy numbers of PSMA3-AS1 in stable transfectants were determined by ddPCR. Results are expressed as copies/µl (n = 3). **(C)** The stable transfectants were seeded on 96-well plates at 3000 cells/well and cultured for 0 − 72 h. The optical density at 450 nm was monitored to evaluate cell proliferation by a CCK-8 assay (n = 3). **(D)** The stable transfectants seeded on 6-well plates (500 cells/well) and allowed to grow for 2 weeks at 37 °C to form visible colonies. After staining, colony numbers were counted and colony formation rate was calculated using the formula described in Materials and Methods section (n = 3). **(E)** The stable transfectants were harvested and subjected to flow cytometric analysis of cell cycle distribution (n = 3). **(F)** Total proteins were extracted from stable transfectants. The levels of cyclin D1 and CDK were determined by western blotting (n = 3). GAPDH served as an internal control. **(G)** The stable transfectants (5 × 10^5^ cells) were seeded on 6-well plates. After adhering to the plates, the cell monolayer was scratched and further cultured for 24. Wound healing rate (%) was calculated using the formula described in Materials and Methods section (n = 5). **(H)** Cell invasion was evaluated by transwell assay. The numbers of invaded cells were counted in five random fields, and the average numbers of invaded cells were calculated (n = 5). ^*^P < 0.05 and ^**^P < 0.01 compared with sh-NC or vector. Student′s t-test was used to compare two groups

The role of PSMA3-AS1 in GC was then investigated. PSMA3-AS1 was notably downregulated in AGS cells with stable knockdown of PSMA3-AS1, while was upregulated in HGC-27 cells stably overexpressing this lncRNA (Fig. 2A). PSMA3-AS1 knockdown did not affect PSMA3 mRNA and protein levels in AGS cells (Supplementary Figure S1A, B). In addition, stable knockdown of PSMA3-AS1 significantly decreased the copy numbers of PSMA3-AS1 in AGS cells. Conversely, stable overexpression of PSMA3-AS1 significantly increased the copy numbers of PSMA3-AS1 in HGC-27 cells (Fig. 2B). The copy numbers of PSMA3-AS1 in unperturbed conditions in both cell lines were shown in Supplementary Figure S2A. Interestingly, AGS cells with stable knockdown of PSMA3-AS1 showed a slower proliferation (Fig. 2C) and a weaker colony formation capacity (Fig. 2D) than the sh-NC group. A higher percentage of G1-phase cells and fewer cells in S- and G2-phases were also observed after stable PSMA3-AS1 knockdown (Fig. 2E). HGC-27 cells stably overexpressing this lncRNA exhibited significant promoting effects on proliferation, colony formation, and cell cycle progression. AGS cells with stable knockdown of PSMA3-AS1 expressed remarkably decreased, while HGC-27 cells stably overexpressing this lncRNA had increased levels of cyclin D1 and CDK6, compared with the sh-NC group and vector group, respectively (Fig. 2F). Additionally, migration (Fig. 2G) and invasion capabilities (Fig. 2H) were restrained after stable PSMA3-AS1 knockdown in AGS cells. After stably overexpressing this lncRNA in HGC-27 cells, we observed enhanced migration and invasion capacities.

### PSMA3-AS1 knockdown accelerates apoptosis of GC cells

**Fig. 3:**
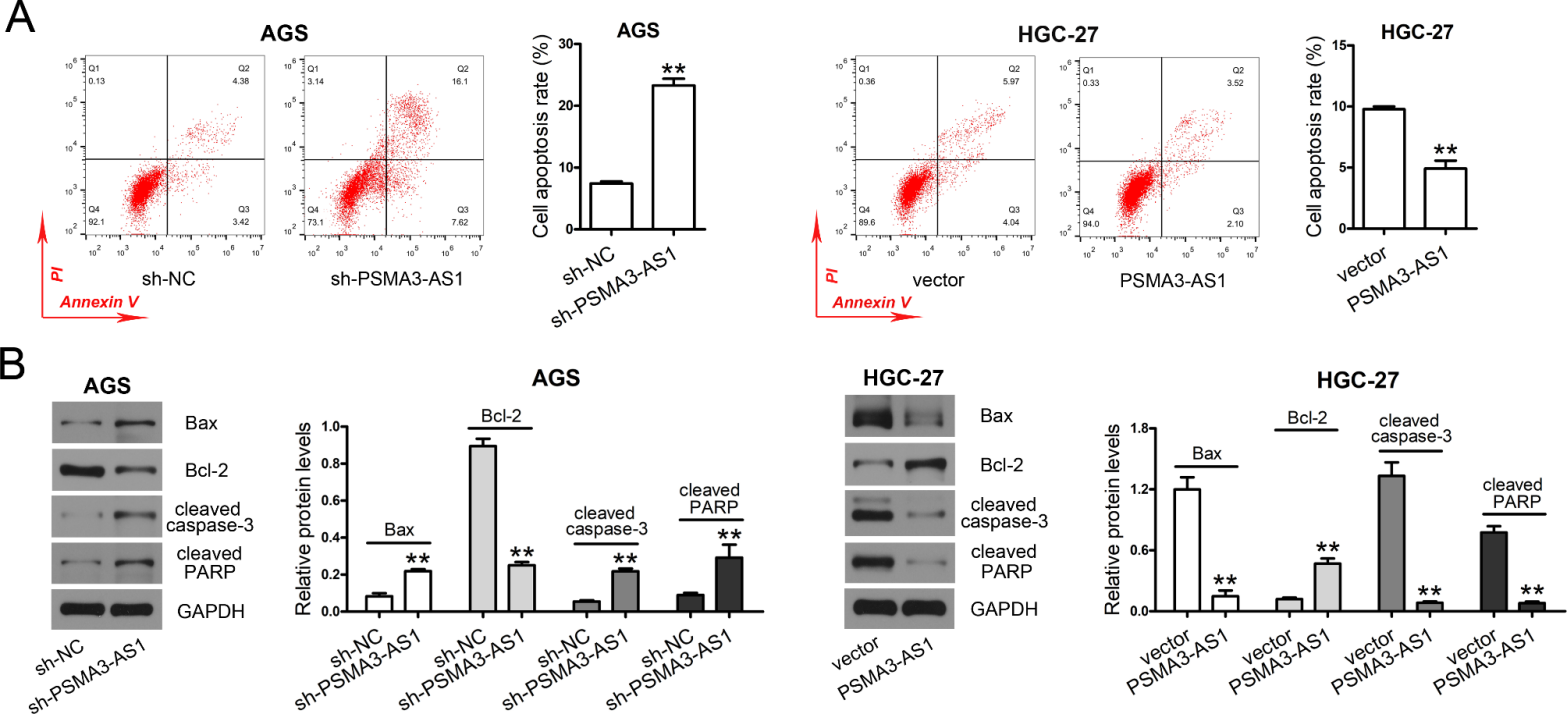
PSMA3-AS1 knockdown promotes GC cell apoptosis. **(A)** Cell apoptosis was evaluated by flow cytometry (n = 3). The apoptotic rate was determined by counting early (Annexin V^+^/PI^–^) and late apoptotic rates (Annexin V^+^/PI^+^). **(B)** Total proteins were extracted from stable transfectants. The levels of Bax, Bcl-2, cleaved caspase-3, and cleaved PARP in AGS and HGC-27 cells were determined by western blotting (n = 3). GAPDH served as an internal control. ^**^P < 0.01 compared with sh-NC or vector. Student′s t-test was used to compare two groups

Stable PSMA3-AS1 knockdown greatly elevated the apoptotic rate of AGS cells (Fig. 3A), upregulated pro-apoptotic protein levels (Bax, cleaved caspase-3, and cleaved PARP), and downregulated anti-apoptotic Bcl-2 levels (Fig. 3B) in AGS cells compared with the sh-NC group. Conversely, stable overexpression of this lncRNA resulted in a reduction in the apoptotic rate of HGC-27 cells compared with the vector group, along with a downregulation of pro-apoptotic regulator and an upregulation of anti-apoptotic regulator.

### PSMA3-AS1 knockdown induces oxidative stress in GC cells

**Fig. 4:**
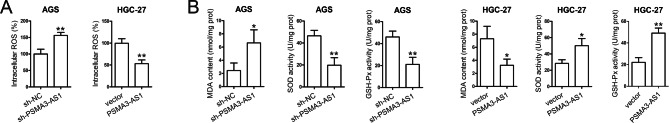
PSMA3-AS1 knockdown activates oxidative stress in GC cells. **(A)** Intracellular ROS level was determined using DCFH-DA fluorescent probes, and the results were read on a fluorescence plate reader (n = 3). **(B)** MDA content, SOD activity, and GSH-Px activity were examined (n = 3). MDA content is expressed as nmol/mg prot. SOD activity is expressed as U/mg prot. GSH-Px activity is expressed as U/mg prot. ^*^P < 0.05 and ^**^P < 0.01 compared with sh-NC or vector. Student′s t-test was used to compare two groups

Next, we investigated the effect of PSMA3-AS1 on oxidative stress. Interestingly, intracellular ROS level (Fig. 4A) and MDA content were significantly elevated, while the activities of SOD and GSH-Px (Fig. 4B) were reduced in AGS cells with stable knockdown of PSMA3-AS1 compared to those in the sh-NC group. HGC-27 cells stably overexpressing this lncRNA had lower levels of intracellular ROS and MDA, and greater activities of SOD and GSH-Px than the vector group.

### PSMA3-AS1 knockdown inhibits the Nrf2 signaling in GC cells

Immunofluorescence assay was performed to detect the activation of the Nrf2 signaling. As shown in Supplementary Figure S3, stable knockdown of PSMA3-AS1 impaired Nrf2 translocation from cytosol to nucleus in AGS cells. Conversely, stably overexpressing this lncRNA promoted nuclear translocation of Nrf2 in HGC-27 cells.

### PSMA3-AS1 acts as a mir-329-3p sponge

**Fig. 5:**
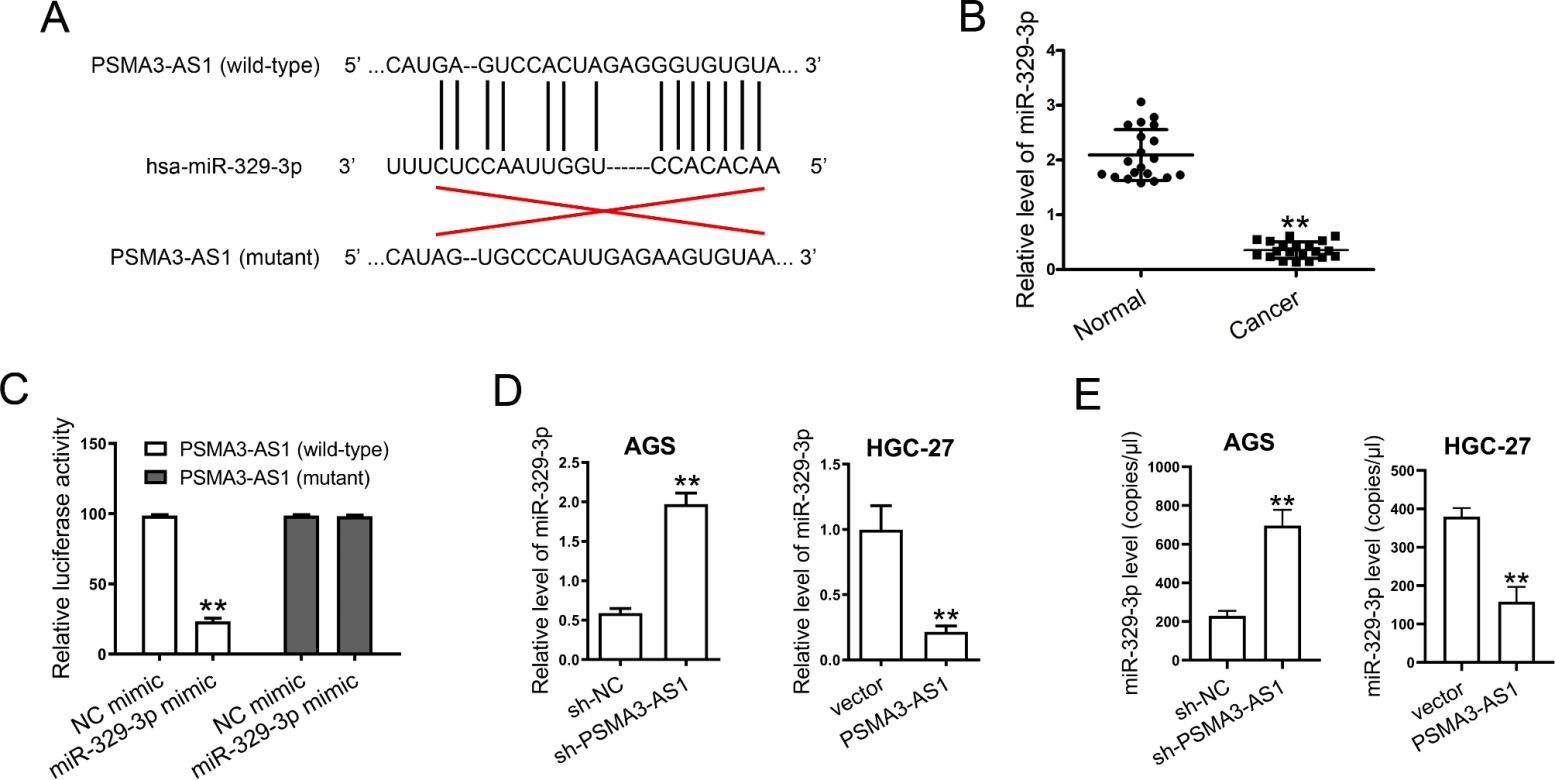
PSMA3-AS1 contains a miR-329-3p binding site. **(A)** The potential binding site for miR-329-3p in PSMA3-AS1 (site 1) was predicted by bioinformatics analysis. **(B)** Total RNAs were isolated from fresh clinical specimens. MiR-329-3p levels in 20 paired clinical specimens were examined by real-time PCR (n = 20). U6 served as an internal control. **(C)** The cells were co-transfected with miR-329-3p mimic or NC mimic and wild-type or mutant PSMA3-AS1 (site 1). The binding of miR-329-3p to PSMA3-AS1 was verified by a dual-luciferase reporter assay (n = 3). **(D)** MiR-329-3p levels in stable transfectants were examined by real-time PCR (n = 3). U6 served as an internal control. **(E)** The copy numbers of miR-329-3p in stable transfectants were determined by ddPCR. Results are expressed as copies/µl (n = 3). ^**^P < 0.01 compared with Normal, sh-NC, vector, or NC mimic. Student′s t-test was used to compare two groups, and one-way ANOVA with Tukey′s post-hoc test was used to compare multiple groups

Bioinformatics prediction showed that PSMA3-AS1 contained two specific binding sites for miR-329-3p (Fig. 5A, Supplementary Figure S4A). Interestingly, miR-329-3p level was lowly expressed in GC tissues (Fig. 5B). Here, we predicated two potential miR-329-3p binding sites in PSMA3-AS1. But we demonstrated that miR-329-3p bound to one of the two sites. The positive result of dual-luciferase reporter assay was shown in Fig. 5C, while the negative result was shown in Supplementary Figure S4B. As shown in Fig. 5C, miR-329-3p mimic significantly reduced the luciferase activity of wild-type PSMA3-AS1 (site 1), but not the mutant type. MiR-329-3p mimic transfection had no effect on the luciferase activity of wild-type or mutant PSMA3-AS1 (site 2; Supplementary Figure S4B). In addition, miR-329-3p level greatly increased in AGS cells with stable PSMA3-AS1 knockdown, while decreased in HGC-27 cells stably overexpressing this lncRNA (Fig. 5D). Stable PSMA3-AS1 knockdown significantly increased the copy numbers of miR-329-3p in AGS cells. Conversely, stable PSMA3-AS1 overexpression significantly decreased the copy numbers of miR-329-3p in HGC-27 cells (Fig. 5E). The copy numbers of miR-329-3p in unperturbed conditions in both cell lines were shown in Supplementary Figure S2B.

### PSMA3-AS1 knockdown exhibits an anti-tumor function in GC cells by regulating miR-329-3p

**Fig. 6:**
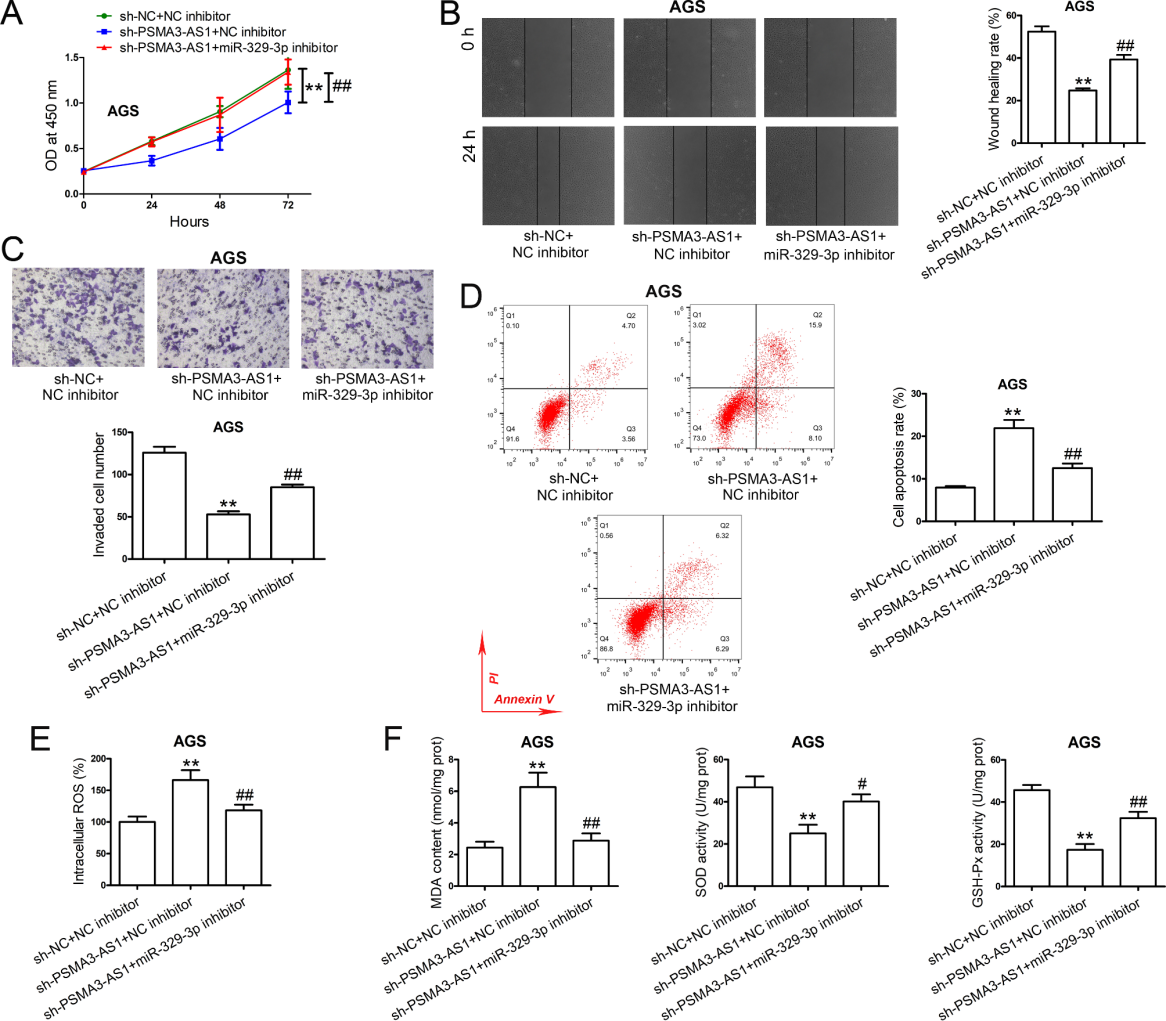
PSMA3-AS1 knockdown exerts its anti-tumor function in GC cells by regulating miR-329-3p. **(A)** AGS cells stably knocked down PSMA3-AS1 were transfected with miR-329-3p inhibitor or NC inhibitor. Cell proliferation capacity was measured by CCK-8 assay (n = 3). **(B)** After transfection, cell migration ability was evaluated by wound healing assay (n = 5). **(C)** After transfection, cell invasion ability was assessed by transwell invasion assay (n = 5). **(D)** After transfection, cell apoptosis was evaluated (n = 3). **(E)** After transfection, intracellular ROS level was examined using DCFH-DA fluorescent probes (n = 3). **(F)** After transfection, MDA content, SOD activity, and GSH-Px activity were measured (n = 3). MDA content is expressed as nmol/mg prot. SOD activity is expressed as U/mg prot. GSH-Px activity is expressed as U/mg prot. ^**^P < 0.01 compared with sh-NC + NC inhibitor. ^#^*P* < 0.05 and ^##^*P* < 0.01 compared with sh-PSMA3-AS1 + NC inhibitor. One-way ANOVA with Tukey′s post-hoc test was used to compare multiple groups

To investigate whether miR-329-3p can mediate the effect of PSMA3-AS1 in GC, AGS cells with stable PSMA3-AS1 knockdown were transfected with miR-329-3p inhibitor or NC inhibitor. Transfection with miR-329-3p inhibitor partially rescued the effects of stable PSMA3-AS1 knockdown on cell proliferation (Fig. 6A), migration (Fig. 6B), invasion (Fig. 6C), apoptosis (Fig. 6D), and oxidative stress (Fig. 6E, F) in AGS cells.

### MiR-329-3p directly targets ALDOA to exert its anti-tumor activity

**Fig. 7:**
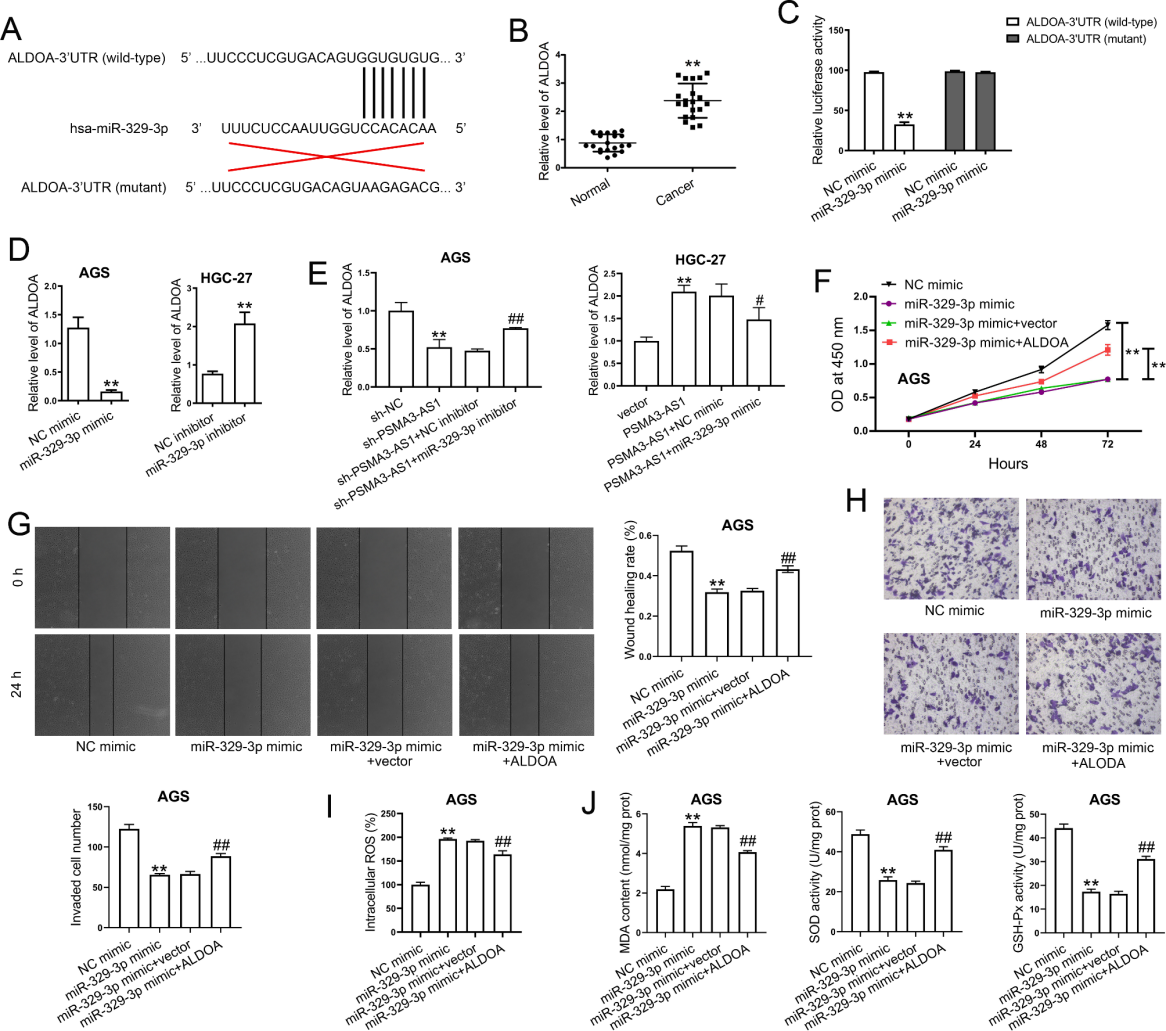
ALDOA is a target of miR-329-3p. **(A)** The potential binding site for miR-329-3p in ALDOA-3′UTR was predicted. **(B)** Total RNAs were isolated from fresh clinical specimens. ALDOA levels in 20 paired clinical specimens were determined by real-time PCR (n = 20). GAPDH served as an internal control. **(C)** The cells were co-transfected with miR-329-3p mimic or NC mimic and wild-type or mutant ALDOA-3′UTR. The binding of miR-329-3p to ALDOA-3′UTR was verified by a dual-luciferase reporter assay (n = 3). **(D)** The cells were transfected with miR-329-3p mimic or inhibitor, and then ALDOA levels were assessed by real-time PCR (n = 3). GAPDH served as an internal control. **(E)** AGS cells stably knocked down PSMA3-AS1 were transfected with miR-329-3p inhibitor or NC inhibitor. HGC-27 cells stably overexpressing PSMA3-AS1 were transfected with miR-329-3p mimic or NC mimic. ALDOA levels in AGS cells were assessed by real-time PCR. GAPDH served as an internal control. **(F)** After transfection, cell proliferation capacity was measured by CCK-8 assay (n = 3). **(G)** After transfection, cell migration ability was evaluated by wound healing assay (n = 5). **(H)** After transfection, cell invasion ability was assessed by transwell invasion assay (n = 5). **(I)** After transfection, intracellular ROS level was examined using DCFH-DA fluorescent probes (n = 3). **(J)** After transfection, MDA content, SOD activity, and GSH-Px activity were measured (n = 3). MDA content is expressed as nmol/mg prot. SOD activity is expressed as U/mg prot. GSH-Px activity is expressed as U/mg prot. ^**^P < 0.01 compared with Normal, NC mimic, NC inhibitor, sh-NC, or vector. ^#^*P* < 0.05 and ^##^*P* < 0.01 compared with sh-PSMA3-AS1 + NC inhibitor or miR-329-3p mimic + vector. Student′s t-test was used to compare two groups, and one-way ANOVA with Tukey′s post-hoc test was used to compare multiple groups

Next, we predicated a miR-329-3p binding site in ALDOA-3′UTR using online bioinformatics tools (Fig. 7A). In addition, ALDOA level was markedly higher in GC tissues (Fig. 7B). The luciferase activity of ALDOA-3′UTR (wild-type) was greatly reduced by the miR-329-3p mimic, whereas had no effect on mutant type (Fig. 7C). In addition, miR-329-3p mimic transfection significantly decreased ALDOA mRNA levels in AGS cells compared with the NC mimic group. Conversely, miR-329-3p inhibitor transfection increased ALDOA levels in HGC-27 cells compared with the NC inhibitor group (Fig. 7D). We then performed more experiments to verify the effect of PSMA3-AS1 on ALDOA was effectively mediated by sponging miR-329-3p. We found that stable PSMA3-AS1 knockdown significantly decreased ALDOA levels in AGS cells, whereas miR-329-3p inhibitor transfection partially rescued the downregulation of ALDOA induced by PSMA3-AS1 knockdown. In HGC-27 cells, PSMA3-AS1 overexpression significantly increased ALDOA levels. MiR-329-3p mimic transfection partially reversed the upregulation of ALDOA induced by stable PSMA3-AS1 overexpression (Fig. 7E). In addition, miR-329-3p overexpression significantly inhibited cell proliferation/migration/invasion (Fig. 7F–H), increased ROS generation (Fig. 7I) and MDA content, and reduced SOD and GSH-Px activities (Fig. 7J) in AGS cells. Whereas, the effects of miR-329-3p overexpression were partially reversed by ALDOA overexpression.

### ALDOA is involved in the anti-tumor role of PSMA3-AS1 knockdown in GC cells

**Fig. 8:**
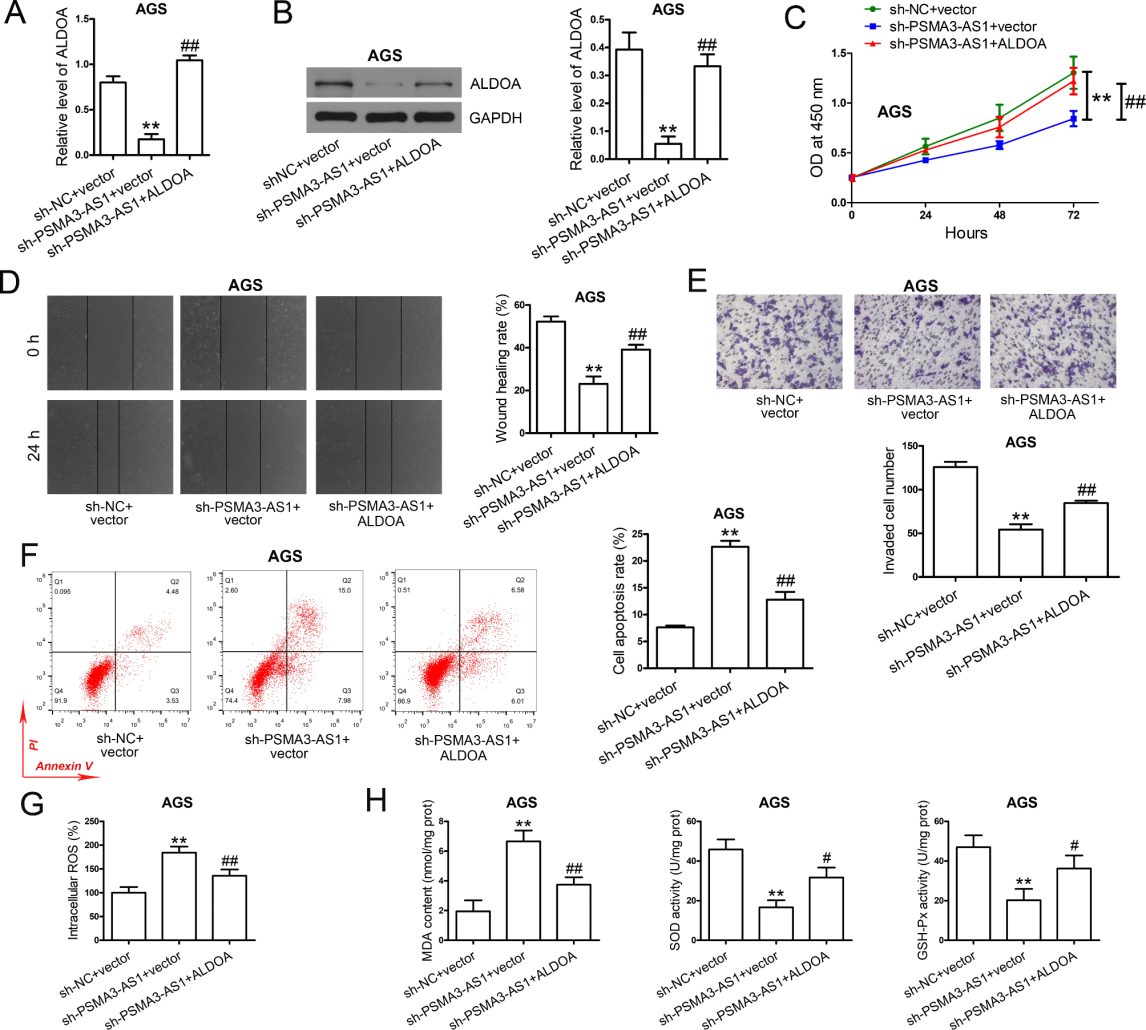
PSMA3-AS1 knockdown exerts its anti-tumor function in GC cells by targeting ALDOA. **(A)** AGS cells stably knocked down PSMA3-AS1 were transfected with ALDOA overexpression plasmid or vector. Real-time PCR was performed to determine ALDOA expression in AGS cells (n = 3). GAPDH served as an internal control. **(B)** After transfection, total proteins were extracted, and western blotting was conducted to determine ALDOA expression in AGS cells (n = 3). GAPDH served as an internal control. **(C)** After transfection, cell proliferation was evaluated by CCK-8 assay (n = 3). **(D)** After transfection, cell migration was assessed by wound healing assay (n = 5). **(E)** After transfection, cell invasion was assessed by transwell assay (n = 5). **(F)** After transfection, cell apoptosis was evaluated by Annexin V-FITC/PI assay (n = 3). **(G)** After transfection, intracellular ROS level was analyzed. **(H)** After transfection, MDA content, SOD activity, and GSH-Px activity were determined (n = 3). ^**^P < 0.01 compared with sh-NC + vector. ^#^*P* < 0.05 and ^##^*P* < 0.01 compared with sh-PSMA3-AS1 + vector. One-way ANOVA with Tukey′s post-hoc test was used to compare multiple groups

To investigate whether PSMA3-AS1 can regulate the biological behavior of GC cells via ALDOA, AGS cells with stable PSMA3-AS1 knockdown were transiently transfected with ALDOA overexpression plasmid or empty vector, and then subjected to subsequent experiments. Stable knockdown of PSMA3-AS1 markedly downregulated ALDOA level in AGS cells, which was reversed by transfection with ALDOA overexpression plasmid (Fig. 8A, B). Interestingly, transfection with ALDOA overexpression plasmid partially reversed the effects of stable knockdown of PSMA3-AS1 on cell proliferation (Fig. 8C), migration (Fig. 8D), invasion (Fig. 8E), apoptosis (Fig. 8F), and oxidative stress (Fig. 8G, H) in AGS cells.

### PSMA3-AS1 knockdown suppresses tumor growth in vivo

**Fig. 9:**
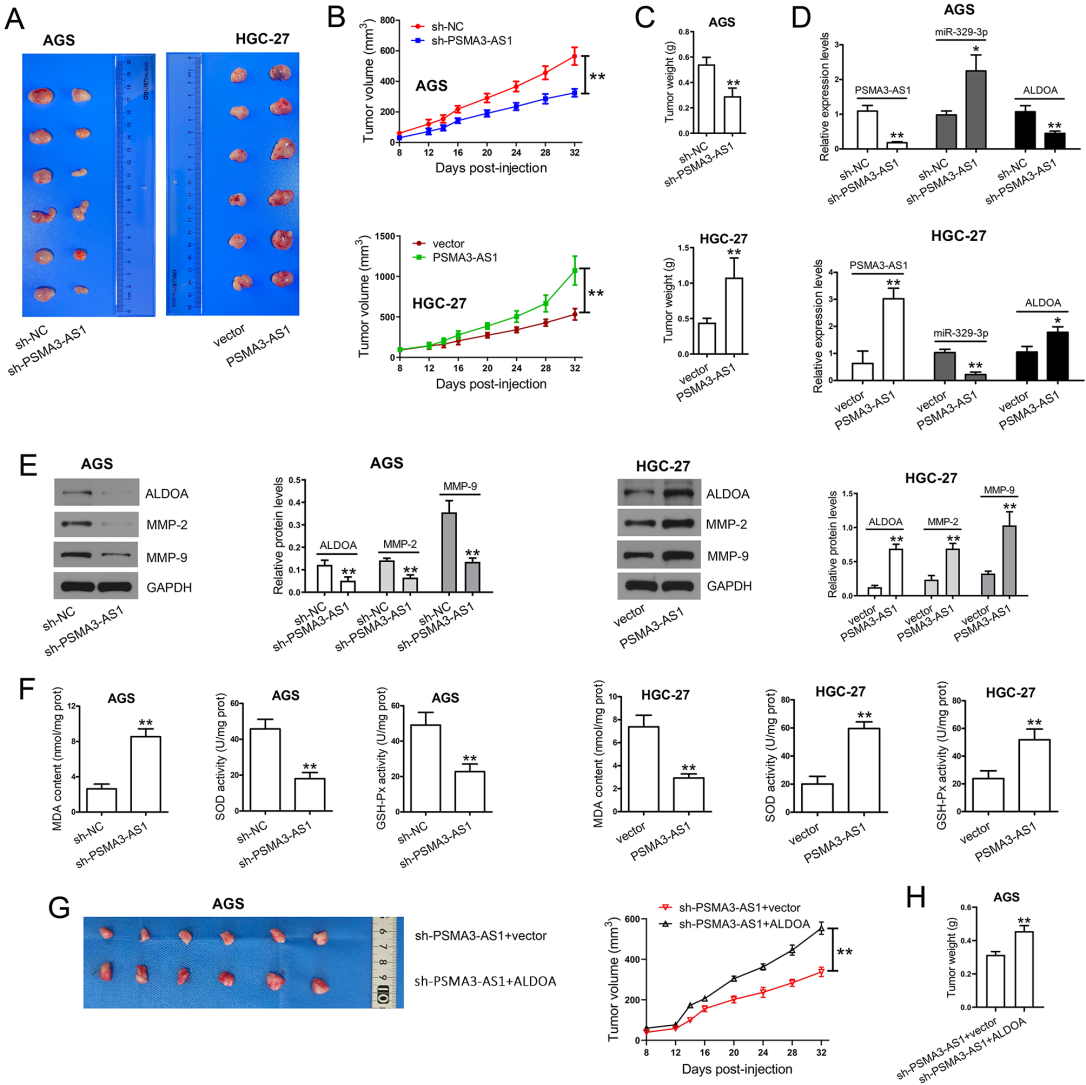
PSMA3-AS1 knockdown inhibits tumor growth in nude mice. **(A)** The nude mice were subcutaneously injected with 1 × 10^6^ stable transfectants. On day 32, the mice were euthanatized. The tumors were excised and photographed. **(B)** Tumor volume was monitored every 4 days (n = 6). **(C)** The tumors were weighed (n = 6). **(D)** Total RNAs were isolated from tumor tissues. PSMA3-AS1, miR-329-3p, and ALDOA levels in tumor tissues were determined by real-time PCR (n = 3). GAPDH was used as an internal control for lncRNA and mRNA, and U6 was for miRNA measurement. **(E)** Total proteins were extracted from tumor tissues. ALDOA, MMP-2, and MMP-9 in tumor tissues were measured by western blotting (n = 3). GAPDH served as an internal control. **(F)** MDA content, SOD activity, and GSH-Px activity in tumor tissues were measured (n = 3). MDA content is expressed as nmol/mg prot. SOD activity is expressed as U/mg prot. GSH-Px activity is expressed as U/mg prot. **(G)** The nude mice were subcutaneously injected with 1 × 10^6^ stable transfectants. On day 32, the mice were euthanatized. The tumors were excised and photographed. Tumor volume was monitored every 4 days (n = 6). **(H)** The tumors were weighed (n = 6). ^*^P < 0.05 and ^**^P < 0.01 compared with sh-NC or vector. Student′s t-test was used to compare two groups, and one-way ANOVA with Tukey′s post-hoc test was used to compare multiple groups

The role of PSMA3-AS1 in tumor growth was evaluated. Injection of AGS cells with stable PSMA3-AS1 knockdown significantly decreased tumor volume (Fig. 9A, B) and tumor weight (Fig. 9C) in nude mice compared with the sh-NC group. The nude mice injected with HGC-27 cells stably overexpressing PSMA3-AS1 showed larger tumor volume and heavier tumor weight than those in the vector group. PSMA3-AS1 and ALDOA levels in the sh-PSMA3-AS1 group were notably downregulated, while miR-329-3p was upregulated compared to those in the sh-NC group (Fig. 9D). As expected, PSMA3-AS1 and ALDOA levels were markedly elevated, while miR-329-3p was reduced after stably overexpressing this lncRNA compared to those in the vector group. The changes in ALDOA levels were further verified by western blotting (Fig. 9E). After stable knockdown of PSMA3-AS1, reductions in MMP-2/-9 levels were detected in tumor tissues. We also observed stable knockdown of PSMA3-AS1 significantly increased MDA content, and reduced SOD and GSH-Px activities (Fig. 9F). Conversely, stably overexpressing PSMA3-AS1 elevated the levels of MMPs and inhibited oxidative stress. Next, we performed more experiments to verify the impact of the PSMA3-AS1-ALDOA axis on tumor growth in vivo. The result showed that ALDOA overexpression partially rescued the inhibitory effect of PSMA3-AS1 knockdown on tumor volume (Fig. 9G) and weight (Fig. 9H) in nude mice.

## Discussion

Our study aimed to explore the function of PSMA3-AS1 in GC progression via loss- and gain-of-function experiments, and investigated the possible molecular mechanism. In the present study, PSMA3-AS1 was overexpressed in GC tissues. Additionally, PSMA3-AS1 affected cell behaviors through regulating the miR-329-3p/ALDOA axis. This study revealed the role and molecular mechanism of PSMA3-AS1 in GC development.

LncRNAs are reported to participate in proliferation, metastasis, cancer metabolic reprogramming, and differentiation, serving as tumor suppressors and oncogenes [23]. PSMA3-AS1 is highly expressed in numerous human cancers [11, 13, 24]. A high level of PSMA3-AS1 is associated with poor prognosis in lung cancer and esophageal cancer [10, 11]. In addition, a tumor-promoting effect of PSMA3-AS1 has also been demonstrated in other human cancers, e.g. cholangiocarcinoma, bladder cancer, and ovarian cancer [13, 25, 26]. The subcellular localization of lncRNAs correlates with their biological function [27]. Previous studies have reported that PSMA3-AS1 mainly locates in the cytoplasm of several types of cancer cells and exerts its function through ceRNA patterns [24–26, 28]. Other evidence has also revealed that PSMA3-AS1 is highly expressed and functions as an oncogene in many cancer types [9–13]. Additionally, PSMA3-AS1 could promote cancer development by functioning as a ceRNA via sponging miR-411-3p, miR-378a-3p, and miR-4504 [11, 13, 14]. However, the expression and function of PSMA3-AS1 in GC are still unclear. We found that PSMA3-AS1 was significantly higher in GC tissues, suggesting a possible role of PSMA3-AS1 in GC progression. Since PSMA3-AS1 is an anti-sense transcript for the gene PSMA3, we thus measured PSMA3 levels in AGS cells with stable PSMA3-AS1 knockdown. We found that PSMA3-AS1 knockdown did not affect the mRNA and protein levels of PSMA3 in AGS cells. Furthermore, PSMA3-AS1 knockdown markedly suppressed cell proliferation, migration, and invasion in vitro, as well as inhibition of tumor growth in vivo; whereas, overexpression of PSMA3-AS1 possessed a tumor-promoting role in GC, which is consistent with previous findings [13, 24]. The finding suggests that PSMA3-AS1 may be an oncogene in GC. Bioinformatics analysis revealed that PSMA3-AS1 contained a miR-329-3p binding site, and we further validated their relationship using the dual-luciferase reporter assay. Additionally, miR-329-3p was decreased in GC cancers and its expression was negatively regulated by PSMA3-AS1. Furthermore, miR-329-3p inhibitor partially reversed the tumor-suppressive role of PSMA3-AS1 knockdown. The finding suggests that PSMA3-AS1 may promote GC progression by modulating miR-329-3p.

Oxidative stress is defined as an imbalance between oxidants and antioxidants, and it plays a crucial role in the pathology of multiple diseases, including type 2 diabetes, Alzheimer′s disease, idiopathic pulmonary fibrosis, and atherosclerosis [29]. Moreover, oxidative stress also participates in the pathogenesis of gastrointestinal mucosal diseases, including GC [29, 30]. Low levels of ROS could promote tumorigenesis, while high ROS production had a tumor-suppressive effect [31, 32]. In a study by Chen et al., celastrol suppressed GC cancer progression by increasing ROS and modulating an antioxidant enzyme, peroxiredoxin-2 [33]. Another study reported that 1α,25(OH)_2_D_3_ increased sensitivity to radiotherapy via the NADPH oxidase/ROS axis in lung cancer and ovarian cancer [34]. However, whether PSMA3-AS1 can affect oxidative stress in GC development remains unknown. In the present study, PSMA3-AS1 knockdown significantly enhanced cell apoptosis and oxidative stress, as evidenced by increased levels of intracellular ROS and MAD, and reduced activities of SOD and GSH-Px. PSMA3-AS1 overexpression inhibited apoptosis and oxidative stress. This result is consistent with previous studies [33, 35]. The Nrf2/HO-1 signaling confers protection against oxidative stress [36]. Mounting evidence has implicated that activation of the Nrf2 signaling is associated with resistance to therapy and cancer progression [37, 38]. Furthermore, studies using Nrf2-deficient mice revealed that Nrf2 was essential for oncogene-induced lung tumorigenesis [39]. Targeting Nrf2 has emerged as a promising strategy for cancer treatment [38, 40]. Our study found that PSMA3-AS1 knockdown greatly inhibited, while PSMA3-AS1 overexpression activated the Nrf2 pathway. The finding suggests that PSMA3-AS1 may affect GC progression by regulating oxidative stress and the Nrf2 signaling.

ALDOA is an enzyme essential for glycolysis and glucose homeostasis [41], and it is highly expressed in numerous human cancers, including GC [42–45]. High ALDOA expression predicted poor prognosis in GC and other human cancers [45–48]. Silencing ALDOA restrained proliferation, invasion, and EMT in GC [45]. In addition, ALDOA knockdown also conferred a tumor-suppressive effect on cervical adenocarcinoma and non-small cell lung cancer cells [42, 49]. As has been reported previously, several signaling pathways are involved in ALDOA-mediated cancer development [44, 49, 50]. Nevertheless, the targeting relationship between miR-329-3p and ALDOA remains unknown. The present study demonstrated the binding of miR-329-3p to ALDOA-3′UTR and negative regulation of ALDOA expression by miR-329-3p, suggesting that ALDOA may be involved in the regulatory effects of miR-329-3p in GC. Salmena et al. first reported a ceRNA hypothesis in 2011 [27]. LncRNAs exert their regulatory functions by competitively binding to miRNAs and thereby regulating target gene expression [51, 52]. In a recent study, PSMA3-AS1 enhanced esophageal cancer cell proliferation and metastasis via acting as a miR-101 sponge [10]. In glioma, PSMA3-AS1 promoted proliferation by affecting the miR-411-3p/HOXA10 axis [14]. However, whether PSMA3-AS1 affects the cancer progression by acting as a ceRNA in GC is still unclear. We observed that PSMA3-AS1 knockdown significantly decreased ALDOA levels in AGS cells, whereas miR-329-3p inhibitor transfection partially rescued the downregulation of ALDOA induced by PSMA3-AS1 knockdown; conversely, PSMA3-AS1 overexpression significantly increased ALDOA levels in HGC-27 cells, whereas miR-329-3p mimic transfection partially reversed the upregulation of ALDOA induced by PSMA3-AS1 overexpression. These findings indicate that PSMA3-AS1 can regulate ALDOA expression in both GC cell lines by sponging miR-329-3p. Moreover, miR-329-3p overexpression significantly inhibited cell growth/metastasis and induced oxidative stress in AGS cells, which were partially reversed by ALDOA overexpression. The above findings indicate that PSMA3-AS1 sponges miR-329-3p to promote GC cell proliferation and metastasis by upregulating ALDOA. Interestingly, ALDOA overexpression partially rescued the tumor-suppressive role of PSMA3-AS1 knockdown in both GC cells and nude mice. The finding suggests that PSMA3-AS1 functions as a ceRNA to regulate ALDOA by sponging miR-329-3p in GC progression.

## Conclusion

In conclusion, PSMA3-AS1 and ALDOA were highly expressed, while miR-329-3p was lowly expressed in GC tissues. PSMA3-AS1 knockdown significantly suppressed GC progression in vitro and in vivo. PSMA3-AS1 overexpression possessed a tumor-promoting effect. The relationship between PSMA3-AS1 and miR-329-3p was verified, and ALDOA was a direct target of miR-329-3p. MiR-329-3p knockdown or ALDOA overexpression partially reversed the tumor-suppressive effect of PSMA3-AS1 knockdown. The result indicates that PSMA3-AS1 may function as a ceRNA and promote GC progression by targeting the miR-329-3p/ALDOA axis. PSMA3-AS1 may serve as a promising target for GC treatment.

## Electronic supplementary material

Below is the link to the electronic supplementary material.

Supplementary Figure S1. Stable PSMA3-AS1 knockdown did not affect PSMA3 mRNA and protein levels in AGS cells. (A) The stable transfectants were harvested to isolate total RNAs. PSMA3 levels were determined by real-time PCR (n = 3). GAPDH served as an internal control. (B) Total proteins were extracted from stable transfectants. PSMA3 levels were determined by western blotting (n = 3). GAPDH served as an internal control. Student′s t-test was used to compare two groups. ns indicates not significant.

Supplementary Figure S2. The copy numbers of PSMA3-AS1 and miR-329-3p in unperturbed conditions in both cell lines. (A) Both cell lines were collected and then subjected to ddPCR analysis. The copy numbers of PSMA3-AS1 in both cell lines were examined. Results are expressed as copies/µl (n = 3). (B) The copy numbers of miR-329-3p in both cell lines were determined by ddPCR. Results are expressed as copies/µl (n = 3).

Supplementary Figure S3. Stable PSMA3-AS1 knockdown impairs Nrf2 translocation from cytosol to nucleus in AGS cells. The stable transfectants on coverslips were fixed in 4% paraformaldehyde for 20 min and then the subcellular location of Nrf2 was assessed by immunofluorescence staining (n = 3).

Supplementary Figure S4. MiR-329-3p mimic transfection had no effect on the luciferase activity of wild-type or mutant PSMA3-AS1 (site 2). (A) The potential binding site for miR-329-3p in PSMA3-AS1 (site 2) was predicted by bioinformatics analysis. (B) The cells were co-transfected with miR-329-3p mimic or NC mimic and wild-type or mutant PSMA3-AS1 (site 2). The binding of miR-329-3p to PSMA3-AS1 was verified by a dual-luciferase reporter assay (n = 3). Student′s t-test was used to compare two groups. ns indicates not significant.

## Data Availability

All data generated or analyzed in this study are included in this article, and are available from the corresponding author on request.
