# Supplementary figures and images for: Long noncoding RNA PSMA3-AS1 functions as a competing endogenous RNA to promote gastric cancer progression by regulating the miR-329-3p/ALDOA axis
Source: Biol Direct. 2023 Jul 4;18:36. doi: 10.1186/s13062-023-00392-8 (PMC10318671; doi:10.1186/s13062-023-00392-8)

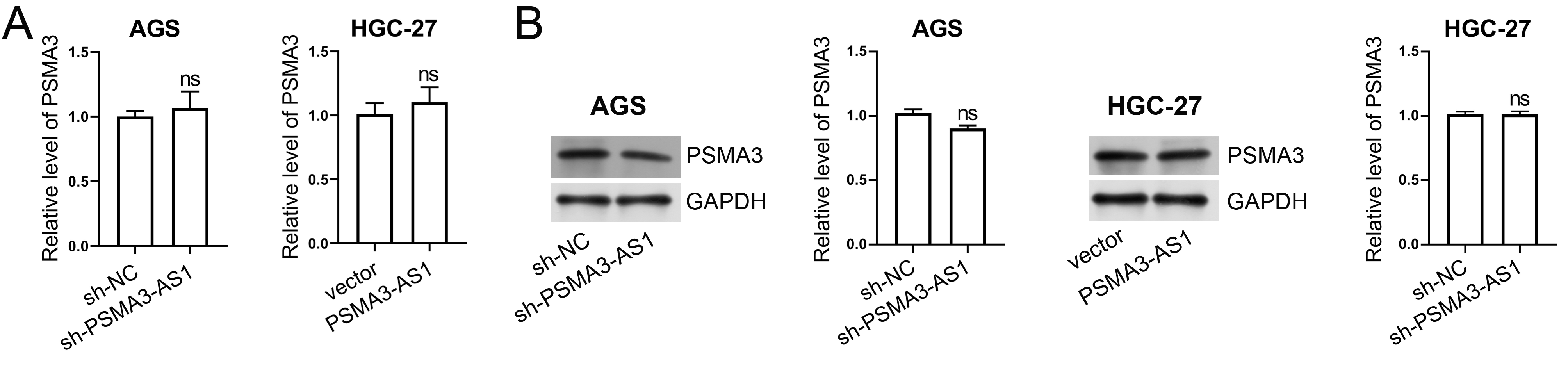

Supplement: Supplementary file 1 — Supplementary Figure S1. Stable PSMA3-AS1 knockdown did not affect PSMA3 mRNA and protein levels in AGS cells. (A) The stable transfectants were harvested to isolate total RNAs. PSMA3 levels were determined by real-time PCR (n = 3). GAPDH served as an internal control. (B) Total proteins were extracted from stable transfectants. PSMA3 levels were determined by western blotting (n = 3). GAPDH served as an internal control. Student′s t-test was used to compare two groups. ns indicates not significant. [file 13062_2023_392_MOESM1_ESM.tif]

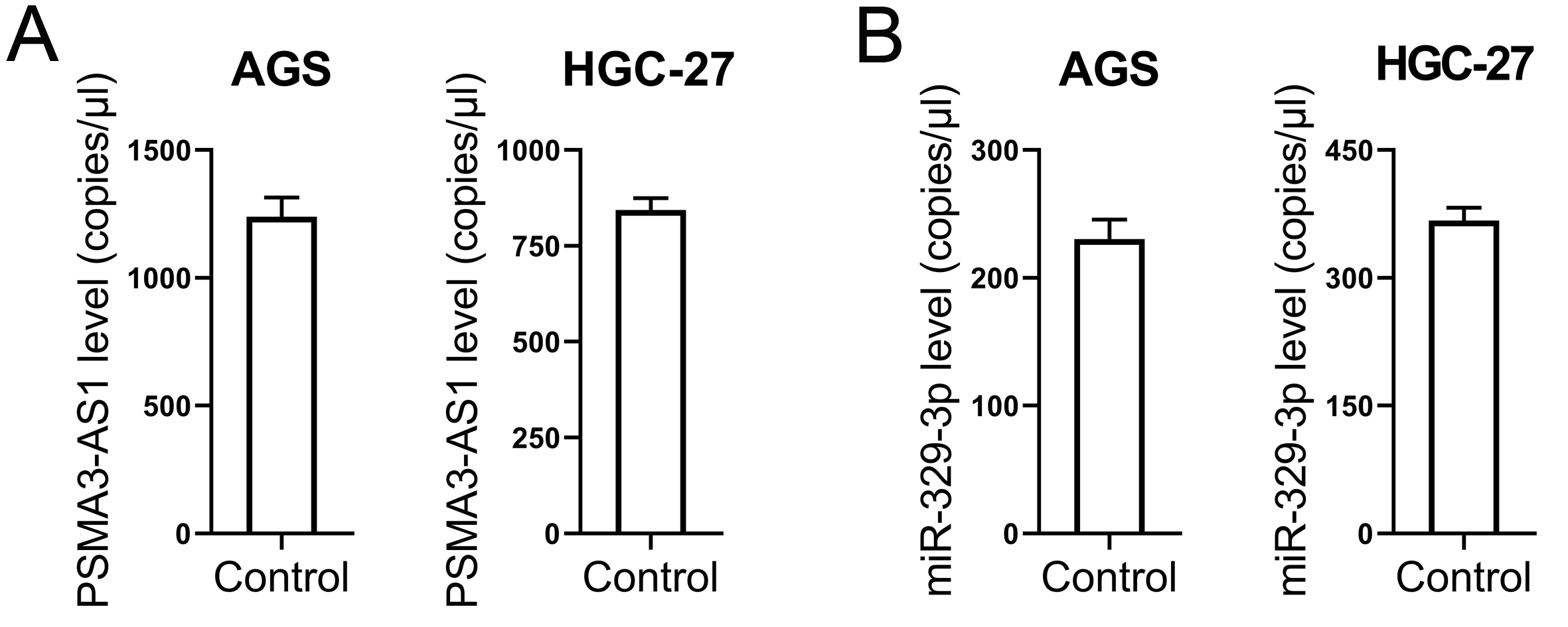

Supplement: Supplementary file 2 — Supplementary Figure S2. The copy numbers of PSMA3-AS1 and miR-329-3p in unperturbed conditions in both cell lines. (A) Both cell lines were collected and then subjected to ddPCR analysis. The copy numbers of PSMA3-AS1 in both cell lines were examined. Results are expressed as copies/µl (n = 3). (B) The copy numbers of miR-329-3p in both cell lines were determined by ddPCR. Results are expressed as copies/µl (n = 3). [file 13062_2023_392_MOESM2_ESM.tif]

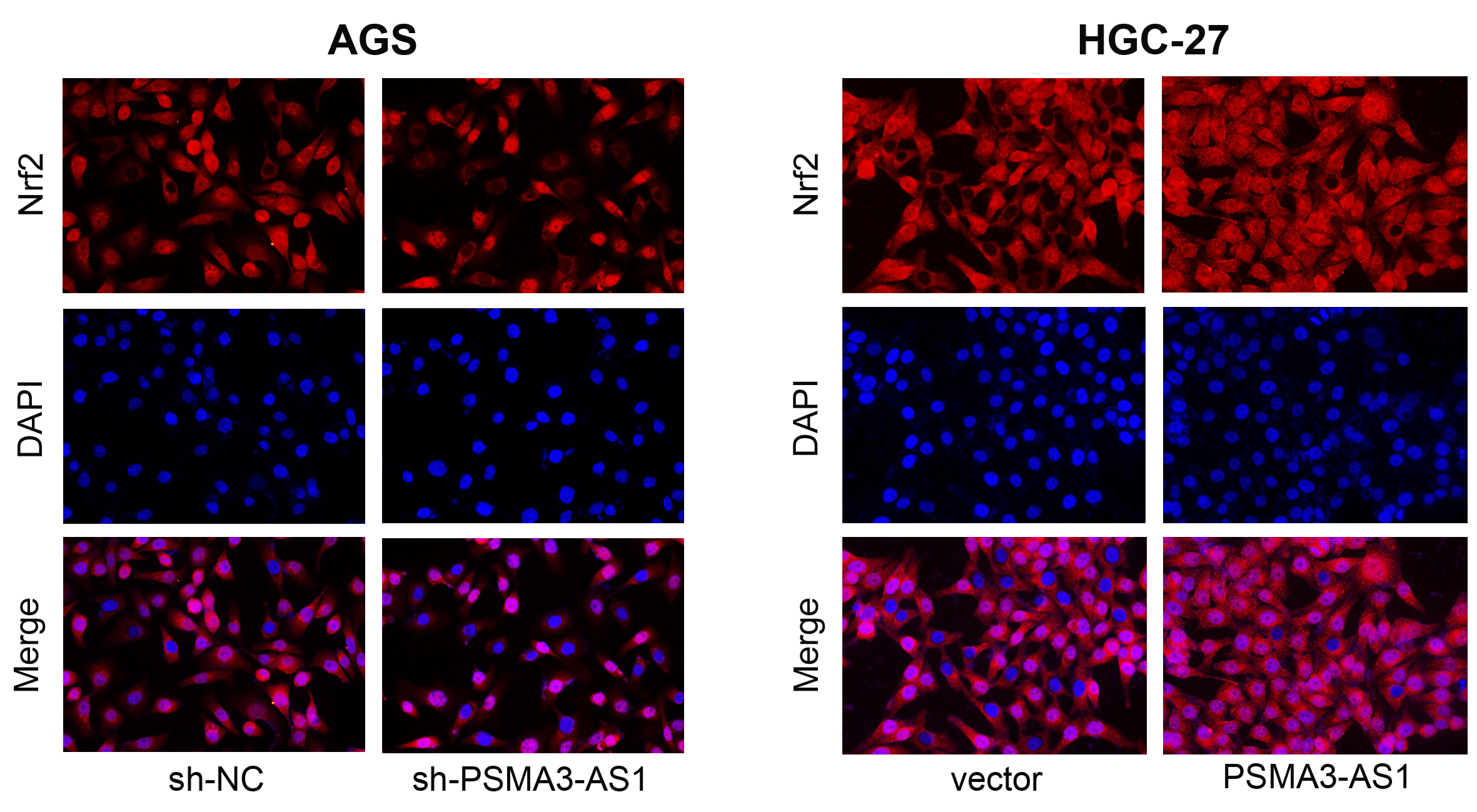

Supplement: Supplementary file 3 — Supplementary Figure S3. Stable PSMA3-AS1 knockdown impairs Nrf2 translocation from cytosol to nucleus in AGS cells. The stable transfectants on coverslips were fixed in 4% paraformaldehyde for 20 min and then the subcellular location of Nrf2 was assessed by immunofluorescence staining (n = 3). [file 13062_2023_392_MOESM3_ESM.tif]

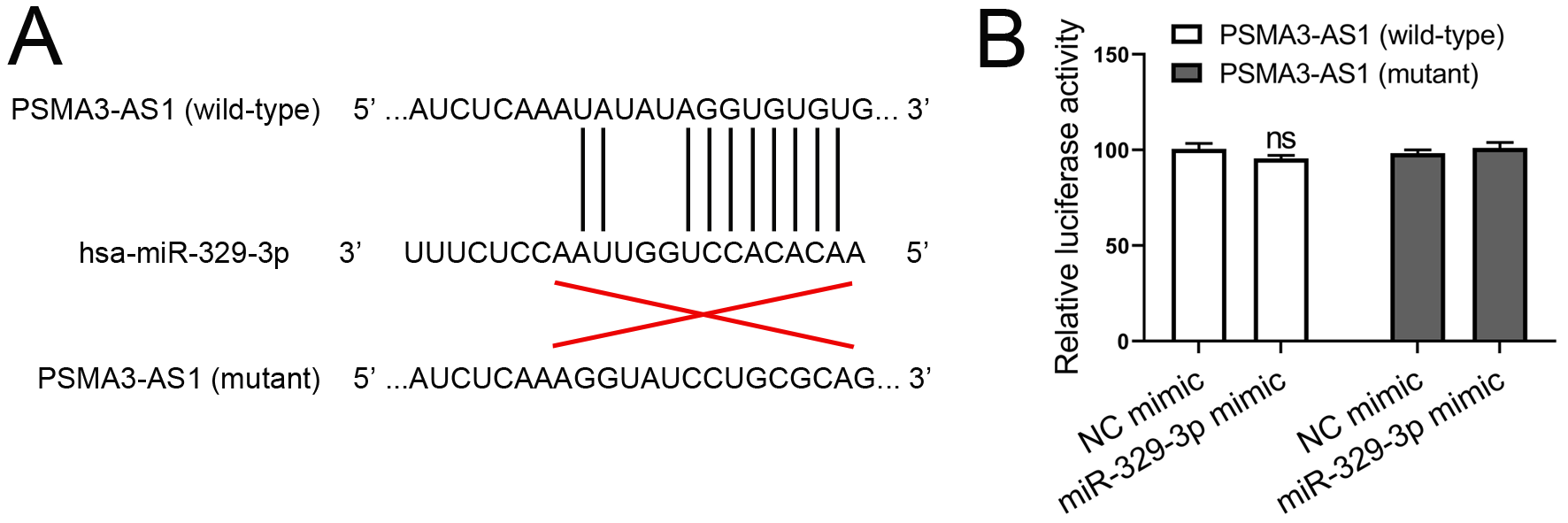

Supplement: Supplementary file 4 — Supplementary Figure S4. MiR-329-3p mimic transfection had no effect on the luciferase activity of wild-type or mutant PSMA3-AS1 (site 2). (A) The potential binding site for miR-329-3p in PSMA3-AS1 (site 2) was predicted by bioinformatics analysis. (B) The cells were co-transfected with miR-329-3p mimic or NC mimic and wild-type or mutant PSMA3-AS1 (site 2). The binding of miR-329-3p to PSMA3-AS1 was verified by a dual-luciferase reporter assay (n = 3). Student′s t-test was used to compare two groups. ns indicates not significant. [file 13062_2023_392_MOESM4_ESM.tif]
